# The Use of Nautical Activities in Formal Education: A Systematic Review

**DOI:** 10.3390/bs13110905

**Published:** 2023-11-03

**Authors:** Daniel Medina-Rebollo, Pedro Sáenz-López Buñuel, Eduardo José Fernández-Ozcorta, Jesús Fernández-Gavira

**Affiliations:** 1 Department of Physical Activity and Sports, Center for University Studies Cardenal Spínola CEU, 41930 Bormujos, Spain; dmedina@ceu.es; 2 Department of Integrated Sciences, University of Huelva, 21004 Huelva, Spain; psaenz@dempc.uhu.es (P.S.-L.B.); eduardo.fernandez@dempc.uhu.es (E.J.F.-O.); 3 Physical Education and Sports Department, University of Seville, 41013 Seville, Spain

**Keywords:** water activities, physical education, primary education, secondary education

## Abstract

Introduction: The implementation of nautical sports, particularly in coastal areas, during the school stage is a growing phenomenon. It offers unique opportunities for students to develop the necessary competencies outlined in their physical education curriculum in an immersive manner, within a natural and inherently motivating environment. Material and methods: This study aims to delve deeper into this subject by conducting a systematic review of the utilization of water sports in formal education. To carry out this bibliographic search, the following keywords were employed: “Nautical Activities”, “Nautical Sports”, “Nautical Camps”, “Formal Education”, “Children Education”, “Primary School”, and “Secondary School”. The Boolean operator “and” was used to combine these keywords during the search conducted in databases such as Scopus, PubMed, Web of Science, and SPORTDiscus. The PRISMA Protocol was utilized for the search process, resulting in the selection and categorization of eight papers into the following thematic areas: Wellbeing, Physical and Mental Health, Education, and Management. Results: The primary findings of this review indicate that the inclusion of these programs within physical education classes enhances overall physical health, mental well-being, and personal development. Conclusions: The results demonstrate a positive impact on peer interaction and short-term improvement in self-confidence.

## 1. Introduction

Childhood is a transformative period during which children undergo physical and mental changes, where cognitive, social, emotional, and physical skills are developed rapidly [1]. In their daily lives, they naturally engage with their surroundings and absorb new experiences like sponges. Building on this concept, new pedagogical models have emerged, which have demonstrated an improvement in motivation in physical education (PE) classes [2]. It is noteworthy to emphasize the adventure education model for activities in natural environments [3]. This model involves designing activities, games, and tasks that allow students to evolve through experiential learning, from which they acquire their knowledge. In this manner, learners first have an experience, then reflect upon it, learn from it, and finally put into practice what they have learned [4]. Specifically, learning is based on the idea that individuals interact with the external environment through their senses and skills [5]. Moreover, we must not overlook the inherent potential of activities conducted in natural environments. In fact, mere contact with nature can be a powerful, exciting, inspiring, educational, and rewarding tool [6].

The nautical activities, in natural environments, can play a crucial role in helping children develop essential skills through motor play [7]. Some authors [8,9] consider that organized aquatic physical activities are highly beneficial during the school years, as they provide opportunities to experience different actions, improve students’ motor skills, and allow for new experiences in a unique environment. On the other hand, other authors [10] mention that activities such as calm-water navigation on vessels like stand-up paddleboarding or canoeing offer children the opportunity to relax and play in a safe setting, without coercive rules or limitations on their capacity for expression and initiative. This significantly facilitates the learning of new skills.

These skills include fostering healthy physical activity habits and enhancing social and cognitive abilities [10]. However, research has shown that children’s interest in such activities varies depending on their developmental stage, life circumstances, and their environment’s disposition toward such activities [10,11]. Therefore, any proposal aimed at incorporating nautical activities should align with children’s specific interests, which evolve as they progress through different stages of childhood. Introducing these activities at younger ages is typically easier, while older children may face competition from factors like screen time consumption, limited schedules, peer influence, and other activities [12]. Consequently, integrating nautical activities into formal physical education classes becomes a strategic approach that, according to various authors [13,14], can potentially contribute to reducing school dropout rates and bridging the achievement gap between students facing academic challenges and those who do not encounter as many difficulties.

Moreover, the importance of the natural environment where nautical activities take place should be emphasized. In a society often detached from the natural world, exposing schoolchildren to wildlife beyond their comfort zones serves an educational, therapeutic, and transcendent purpose. This exposure allows them to experience firsthand the impact of human activities on nature [15,16,17]. Additionally, engaging in nautical activities fosters discipline, teamwork, and a deeper connection with the aquatic environment.

The promotion of active and healthy lifestyles, which have a positive impact on the mental health of schoolchildren, is crucial for their growth and education. In fact, this aspect is already inherent in the education systems of most democratic countries [18]. However, it is important to acknowledge that a significant portion of the population, particularly in highly developed countries, lack sufficient physical activity and instead have sedentary lifestyles [19]. These sedentary habits, combined with improper eating habits, or even mental illnesses, contribute to childhood diseases such as obesity, diabetes, and asthma [20,21,22]. On the contrary, engaging in nautical activities such as sailing increases students’ confidence and competence, key personal and interpersonal skills, life-specific skills, specific navigation skills, physical fitness, and overall mental well-being [23]. This has a significant impact on reducing illnesses in school-aged children and maintaining healthy standard somatotypes.

Amidst this prevailing environment of limited physical activity and minimal contact with the natural world, opportunities arise in the form of water sports activities in aquatic environments. These activities allow many schoolchildren to explore these spaces with curiosity and a sense of discovery, while fostering sportspersonship and physical activity habits that are uniquely possible in “blue environments” [24]. Moreover, these environments represent the largest area of our planet, where the World Health Organization defines health as “a state of complete physical, mental, and social well-being, and not merely the absence of disease or infirmity” [25]. Engaging in sports and connecting with the natural environment and others can contribute to personal well-being [26], and in many cases, it also facilitates, even in informal educational contexts, the decision-making and progressive emancipation of adolescents [27].

But what exactly are water sports? They encompass a wide range of activities, and their classification may vary depending on the organizing body or sports entity. The International Olympic Committee (IOC), International Sailing Federation (ISAF), International Canoe Federation (ICF), and the International Surfing Association (ISA) each offer their own classification systems. However, when examining these sports at the school level, the scientific literature primarily focuses on certain ones such as surfing, sailing, and kayaking, among others [28,29,30].

Nevertheless, these sports share a common characteristic—they are performed in an aquatic environment, often in natural settings, and provide various benefits in the school context, which should be considered.

Some authors have delved into the physical benefits produced by some of the nautical activities. Alecu et al. [10] conducted a program involving canoeing for children aged 8 to 12 years and found significant improvements in motor skills, coordination, and control of major and minor muscle groups. These activities enhance body coordination, muscle volume and tone, and reduce muscle fat. Other researchers, such as Janssen et al. [31], have conducted extensive literature reviews and determined that activities need to be at least moderately intense to produce organic changes, such as lowering blood pressure or reducing obesity in children. Moderate-intensity activities affect the cardiovascular and respiratory systems, leading to the desired improvements.

Therefore, it is crucial for schoolchildren to engage in sports within environments like aquatic settings. The involvement of families and educational institutions, capable of integrating these sports into their curriculum, holds a pivotal position in facilitating participation and harnessing the related advantages [32]. These endeavors should consistently be executed through meticulously organized ‘Sports for All’ programs, spanning across educational institutions and local governing bodies [10].

Following this introduction, the objective is to conduct a systematic review of the utilization of nautical activities in formal education, with the aim for this to be understood according to Coombs and Ahmed [8,33], who define it as “‘the institutionalised, chronologically graded and hierarchically structured education system’—spanning schools and university”. Therefore, in the proposed work, the concept of formal education serves to define the entire process of training and learning imparted by the official educational institutions of a society. This includes, in our case, all compulsory schooling for children and adolescents aged 6 to 16, such as primary and secondary schools.

In adherence to the SMART methodology, the present study posits an inquiry of significant import: to what extent does the inclusion of aquatic activities within the formal educational curriculum contribute to the enhancement of holistic well-being, encompassing physical, social, and mental skill development, and the cultivation of student motivation and engagement among children and adolescents aged 6 to 16, at an international level? In accordance with this research question, the following hypothesis is formulated: “The integration of aquatic activities into the formal educational curriculum is anticipated to exert a substantive and positive impact on the overall health and multifaceted skills (mental, social, and physical) of children and adolescents. Furthermore, it is posited that this integration will serve to catalyze heightened motivation and active participation within the educational milieu”.

## 2. Materials and Methods

This study adhered to the guidelines outlined in the Cochrane Handbook for Systematic Reviews of Interventions [34] and followed the PRISMA Statement [35], ensuring a rigorous and transparent approach to the review process. This review has been registered on OSF (Open Science Framework), specialized software for systematic reviews, with a focus on education, among other searches, and can be accessed via the following link: https://doi.org/10.17605/OSF.IO/TH3KG (accessed on 31 October 2023) [36]. 

### 2.1. Search Strategy

A comprehensive search strategy was developed to identify peer-reviewed journal articles until 1 November 2023, without any exclusion based on publication date. The systematic review focused on studies published primarily or secondarily in English in journals indexed in the PubMed, Scopus, Web of Science databases, and SPORTDiscus. The preliminary search utilized specific keywords, guided by experts in the field. The finalized keywords used for the systematic search were “Nautical Activities”, “Nautical Sports”, “Nautical Camps”, “Formal Education”, “Children Education”, “Primary School”, and “Secondary School”, with the conjunction operator “AND” and “OR” employed to combine them. The study population consisted of school children and adolescents aged 6 to 16 years who had participated in nautical activities linked to their formal education.

### 2.2. Eligibility Criteria

Following the recommendations of Tricco et al. [37], this systematic review encompassed all peer-reviewed conceptual or empirical perspectives. The included studies specifically addressed the implementation of nautical activities in the context of formal education. These encompass both quantitative and qualitative studies published at any time. The inclusion criteria involved studies with participants who were enrolled in school, aged between 6 and 16 years, and engaged in nautical activities within their educational institutions. Conversely, studies were excluded if they met one or more of the following criteria: 1. focused on populations in pre-mandatory education (preschool) or post-mandatory education (baccalaureate); 2. included participants below 6 years of age or above 16 years of age; 3. consisted of editorial letters, systematic reviews, or abstract proceedings; 4. written in a language other than English (at least the abstract); 5. not relevant to the research topic; 6. duplicated previously selected studies; and 7. involved schoolchildren not linked to formal education.

The article selection process was conducted in two stages. The first stage involved screening studies based on abstracts and titles, while the second stage involved a thorough examination of the selected articles to ensure compliance with the inclusion criteria. Two external experts (D.M.R. and J.F.G.) independently conducted the selection and screening process without knowledge of each other’s work. Any discrepancies between the two reviewers were resolved by a third party (E.J.F.-O.), serving as an arbitrator. The articles that did not meet the eligibility criteria were documented using a checklist.

### 2.3. Selection and Search Processes

Based on a systematic review of the literature specifically addressing nautical activities and formal education, following the guidelines proposed by Tricco et al. [37] regarding the inclusion of empirical, conceptual, and peer-reviewed papers, a total of 442 articles were initially identified. Among these, 30 duplicates were identified and subsequently removed. Further evaluation of titles and abstracts led to the exclusion of an additional 363 articles. As a result, 50 articles remained for a detailed review of the full text. After applying the aforementioned exclusion criteria, a total of 8 articles met all the specified criteria.

The exclusion filters were applied based on the following grounds: studies involving populations in pre-school (infant) or post-school (high school) (8 studies); studies involving participants below 6 years of age or above 16 years of age (12 studies); editorial letters, systematic reviews, or abstract procedures (6 studies); works written in languages other than English (3 studies); and studies involving schoolchildren not linked to formal education (13 studies).

The eight selected articles for in-depth analysis that satisfied all the criteria are as follows: [13,23,38,39,40,41,42,43].

### 2.4. Data Extraction Processes for Analysis

The final review included 8 articles, and a table was created (see Table 1) to present and facilitate the comprehension of the following information: (a) general study details, including the title of the work, names of the authors, and year of publication; (b) sample characteristics, such as the number of participants, their age, and gender; (c) tools and instruments utilized for analysis in the study; and (d) intervention specifics, encompassing the authors’ proposed objectives and the obtained results from the intervention.

### 2.5. Review of the Quality of Obtained Articles

To assess methodological quality, various instruments were employed based on the study type. In the case of qualitative studies (see Supplementary Material), the Standards for Reporting Qualitative Research (SRQR) tool [44] was used, comprising 21 items categorized under “title and abstract”, “introduction”, “method”, “discussion”, and “other”. For quantitative studies (see Supplementary Material), the Consolidated Standards of Reporting Trials (CONSORT) checklist, initially designed by Schultz et al. [45] and adapted by other authors [46], was applied. This tool consists of 20 items distributed across the categories of “title and abstract”, “introduction”, “method”, “results”, “discussion”, and “other information”.

Finally, for the mixed-method article [13], an independent joint assessment was conducted using the two aforementioned scales. Each study was individually scored by two reviewers who assessed different sections of the studies and assigned a score of 1 if the study met the criteria and 0 if it did not. Any discrepancies between the reviewers were resolved through a review and discussion of the original study until a consensus was reached (*n* = 2).

## 3. Results

### 3.1. Study Flow

**Figure 1:**
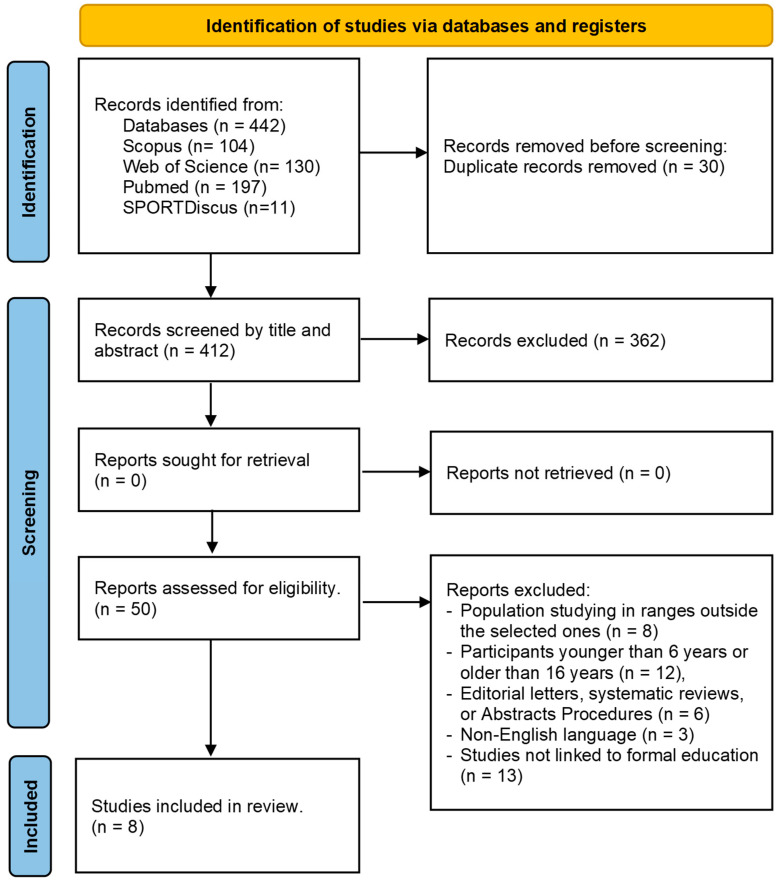
Flowchart of the selection process according to the recommendations of the PRISMA Statement [35]. Source: Own elaboration.

Figure 1 illustrates the flow chart of the present review, depicting the progression from the initial 412 references to the final inclusion of eight articles in the analysis.

Table 1 presents a descriptive overview of the articles, providing essential information such as the study title, author and year, type, sample size, and results. This section is divided by subheadings. It provides a concise and precise description of the experimental results, their interpretation, as well as the experimental conclusions that can be drawn.

### 3.2. Analysis of the Methodological Quality of the Studies

To check the methodological quality of the studies, the analysis of the quality of the four qualitative articles evaluated in the research showed that two articles obtained a high score of 13 points or more out of a total of 21 items, with one article achieving the maximum score of 15 points [23], followed by Schmitt et al. [42] with 13 points. Two qualitative articles, however, were below the threshold of 10 points, namely Machota-Blas [40] with five points, as well as Santos et al. [41], so it could be understood that the quality of their papers is low. However, in a qualitative observation, and regardless of the scale used, the article by Machota-Blas [42] is a descriptive article of a teaching experience that is well presented and documented. In contrast, Santos et al.’s research [41] delineates collaborative efforts across multiple schools in Portugal aimed at crafting a curriculum centered on life skills via the medium of surfing. The study delves into the experiences and insights gained in a manner that mirrors the aforementioned work, offering comprehensive documentation. The resulting implications and potential applicability are unquestionably of exceptional quality and significant relevance within the academic domain.

In relation to the methodological quality of the three quantitative studies evaluated, two articles were obtained with a score of 13 points out of 20 in total [39,43], and another with 16 [38]. Rocher et al.’s study [13], which employed a mixed-method approach, exhibited the highest overall quality in absolute terms, scoring 15 points on the qualitative scale and 18 points on the quantitative scale.

### 3.3. Thematic Analysis: General Description and Primary Themes

After conducting a literature analysis, various thematic areas have emerged to categorize the reviewed articles. These include the following: health [38], physical and mental well-being [13,23,39], education [39,40,41,42], and management [43]. The subsequent section provides a detailed description of each thematic area discussed.

#### 3.3.1. Well-Being, Physical and Mental Health

The article by Bravo et al. [38] examines the significance of incorporating surfing into physical education classes as a means to mitigate the risk of cardiovascular diseases. The study investigated the effects of an eight-week surfing intervention, with one hour of surfing per day, conducted in two secondary schools. The findings indicate that participating students achieved durations and heart rates that align with health recommendations.

In a separate study conducted by Rocher et al. [13], involving nearly five hundred children and adolescents engaged in surfing, rowing, sailing, and canoeing, it was reported that these activities contributed to the improvement of their overall health.

Different authors have extensively investigated the relationships between the significant potential of nautical activities and the improvement of self-esteem and life skill development. Specifically, Cotterill et al. [23] worked with schoolchildren between 9 and 13 years old in light sailing programs and identified specific benefits in terms of physical and mental health, the development of key life skills, and self-esteem. Similarly, Silva et al. [39] conducted research on adolescents engaging in surfing and found that participating in this sport at the school level serves as a socializing agent, facilitating the formation of new friendships and group inclusion, which, in the short term, positively influences self-confidence, aligning with the findings of Rocher et al. [13]. Lastly, Rocher et al. [13], following an extensive study involving over 500 schoolchildren (children and adolescents) in surfing, rowing, sailing, and canoeing, assert that these activities enhance mental health and improve participants’ perception of their overall well-being.

#### 3.3.2. Education

The inclusion of nautical activities in physical education classes can offer various benefits to students. According to Silva et al. [39], the primary advantages include improvements in individual expectations, self-confidence, and peer socialization. In fact, the authors suggest that even with only six weeks of surfing classes integrated into the school curriculum, significant changes are already observed in adolescents.

Additionally, Machota-Blas et al. [40] highlight that the development of teaching units focused on nautical activities in the sea increases adolescents’ interest in marine conservation and provides them with an unparalleled environment to achieve all the educational objectives of this stage.

Authors like Santos et al. [41] emphasize the value beyond the educational aspect, which can only be acquired in an environment that facilitates comprehensive immersion in the natural surroundings. Specifically, they underscore the importance of sports in the natural aquatic environment for the development and transfer of life skills.

Addressing the issue of gender roles in maritime sailing, Schmitt et al. [42] inquire into the biases that favor adolescent boys, who are often considered more qualified and legitimate for leadership roles such as helmsmen, while adolescent girls are predominantly assigned crew positions. The authors reflect on the design of educational programs to ensure that such differences do not occur and to promote equal opportunities.

#### 3.3.3. Management

Segado et al. [43] conducted a study aimed at assessing the loyalty and satisfaction levels of 350 schoolchildren, ranging from 6 to 12 years old. The researchers employed a survey to gauge the participants’ future intention to return to nautical camps. The findings demonstrated that satisfaction and psychological commitment positively influence user loyalty and their future intention to engage in the camps. Moreover, the study revealed that loyalty could only be effectively measured through one specific dimension: word of mouth.

## 4. Discussion

Nautical sports during the school years have been recognized by various authors as an effective means of promoting significant experiential learning in children. This type of learning serves as a fundamental pillar of primary and secondary curricula, as it enables students to acquire essential skills, improve their mental and physical health, enhance overall well-being, and develop specific knowledge related to physical education, such as learning nautical sports, navigation, understanding weather conditions, wind patterns, and more.

In line with the overarching objective of this study, which focuses on conducting a systematic exploration of water sports in the context of school education, it is pertinent to discuss the main findings that warrant further examination; however, we would like to point out, as Cotteril et al. [23], the shortage of studies that link water sports with formal education for children and adolescents.

Regarding the health benefits of these sports, authors such as Bravo et al. [38] and Rocher et al. [13] highlight their positive impact on general physical health, including significant improvements in cardiac health. However, contrasting effects have been observed by other authors, like Torres et al. [47], particularly in swimming and rowing, where exercise-induced asthma can occur.

In relation to well-being and physical and mental health, Rocher et al. [13] found evidence of a correlation between the practice of water sports and improved well-being and mental health among schoolchildren. Similarly, Barton et al. [48] conducted an extensive study involving over 1000 individuals in the United Kingdom, which determined that aquatic activities in marine environments enhance self-esteem, a finding corroborated by Cotterill et al. [23], who noted even greater improvements in younger age groups. Additionally, Lloret et al. [49] emphasized the importance of practicing water sports in well-preserved natural areas, suggesting their potential as preventive and rehabilitative health strategies. Furthermore, authors such as Cavalheiro et al. [50] stressed the significance of implementing these activities during challenging times, such as the COVID-19 pandemic, which has had a substantial impact on mental health.

Considering the importance of connecting with nature to maximize the benefits of water sports, it is crucial to raise awareness among schoolchildren about the potential impact of these activities on the natural environment. Morales-Baños et al. [51] highlight that the natural space itself is the ideal setting for observing and understanding this impact.

Regarding the educational objectives related to teaching physical education in the aquatic environment, Machota-Blas et al. [40] emphasized the unparalleled experiential learning framework provided by nature. Once the common dynamics of these environments have been learnt, students can acquire lifelong learning skills, as noted by Santos et al. [41]. Similarly, Ortizn et al. [52] argued that such work in an aquatic environment should start at an early age, as it offers significant cognitive and motor benefits through comprehensive, integrated, and globalized learning. These learning experiences, combined with play, foster a sense of security, affection, and confidence, thus enhancing students’ self-esteem [53].

The third set of articles found through the systematic search pertains to sports management with schoolchildren in nautical camps. However, only one relevant study was identified, namely the research conducted by Segado et al. [43]. This study focused on measuring the loyalty and satisfaction of schoolchildren participating in nautical camps and their intention to participate in such camps in the future. The findings revealed that satisfaction and psychological commitment positively influence the loyalty or future intention of participants. These results differ from the work of Trespalacios-Gutiérrez et al. [54], who suggest that satisfaction with a product is primarily determined by the convenience of the product and the range of available options. In the context of nautical camps, this could correspond to the type of nautical activities offered to students. Segado et al. [43] determined in their study that loyalty could only be measured based on one dimension, which was word of mouth.

Regarding the implications of promoting participation in nautical activities in formal education, there are several aspects to consider. On the one hand, the positive impact of these activities on students’ health and well-being is highlighted. This suggests a need for an increased emphasis on promoting active and healthy lifestyles through aquatic activities. On the other hand, the implementation of these programs enhances social skills, such as self-confidence and the formation of friendships. It also improves student loyalty and satisfaction with curriculum-related subjects, such as physical education, natural sciences, or social studies. Consequently, it enhances the overall perception of the educational institutions that offer such programs, including schools and high schools.

With regard to future prospects, the research efforts will be specifically focused on exploring the benefits of these activities within formal education. Another area of interest is in investigating the impact of these activities on students from different backgrounds and cultures to determine the cultural factors that may influence them. Another promising line of research, which could be the subject of future studies, is examining how these aquatic activities, whether conducted within formal or informal education settings, influence their close-knit groups, particularly family and friends, as well as their personal and professional expectations.

The study exhibits certain limitations that need to be acknowledged. One notable limitation is the potential exclusion of specific articles due to the extensive and varied nature of nautical activities, including but not limited to surfing, kayaking, dinghy sailing, rowing, and more. The classifications of these activities often rely on different federations or institutions, which may have contributed to an extensive and potentially exhaustive search process. However, it is essential to recognize that the study’s intended scope was not aimed at comprehensively covering all conceivable combinations of terms associated with aquatic activities and education.

Another limitation of this study is that, due to the scarcity of studies found in the systematic review, the results have proven to be highly heterogeneous and cannot be generalized, as the studies addressing the topic have not followed a common criterion. This suggests that this field is still in the early stages of research, which is partly evident due to the limited results mentioned and the recent nature of all the studies (all published within the last nine years).

Moreover, the reference to future research endeavors suggests a potential bias or personal interest, as it mentions the commitment to supporting and furthering the research work of the first author, particularly concerning his doctoral thesis. While personal commitment and enthusiasm for a research area are valuable, it is important to maintain a balanced and objective perspective in academic research. This could be seen as a potential conflict of interest or bias that might affect the interpretation of results and conclusions. Thus, in future research, it is essential to ensure an unbiased approach to data analysis and reporting.

## 5. Conclusions

In conclusion, this systematic review offers a comprehensive overview of the impact of incorporating aquatic activities into formal education for children and adolescents, aged 6 to 16, on an international scale. The research was conducted through the meticulous evaluation of 401 articles, resulting in the careful selection and categorization of eight articles across four distinct thematic areas: well-being, physical and mental health; education; gender equality; and management.

Within the realm of well-being and physical and mental health, the study consistently highlights the positive outcomes of aquatic activities, including surfing, sailing, and rowing, in mitigating cardiovascular risks and enhancing overall health. The alignment of these findings with established health recommendations underscores the advantageous impact of these activities on physical health. Moreover, the research underscores the value of these activities in promoting mental and physical well-being.

In the domain of education, the study emphasizes the significant advantages of integrating nautical activities into formal education. The documented improvements in students’ self-confidence, peer socialization, and their ability to set and attain individual expectations confirm the benefits of these activities in fostering personal and social development. The emphasis on marine conservation and the development of crucial life skills in line with educational objectives underscores the multifaceted advantages of this integration.

The examination of gender equality reveals the importance of providing equal opportunities for both boys and girls in leadership roles within maritime activities. This recognition is of utmost relevance to the overarching aim of enhancing social and skill development, ensuring that all students can benefit equitably from these activities.

In the context of management, the study delves into the loyalty and satisfaction levels of schoolchildren regarding nautical camps, demonstrating that satisfaction and psychological commitment positively influence user loyalty and their future intention to participate in these activities. This underscores the research focus on motivating and engaging students in the educational process.

In summary, this systematic review provides a well-rounded perspective on the international-scale impact of incorporating aquatic activities into formal education. It substantiates the initial hypothesis, confirming that this integration significantly enhances the overall health and skill development of children and adolescents. The systematic approach to literature evaluation and categorization enhances our understanding of the multifaceted benefits of nautical activities in education. This study contributes valuable insights for educators, policymakers, and researchers aiming to promote the holistic development of students through the integration of aquatic activities into formal education.

## Figures and Tables

**Table 1:** Descriptive register of articles.

Study	Type	Thematic Areas	Sample	Results
[38]	Quantitative	Well-being, Physical and Mental Health	24 (20 males), Age: 15–16	High school students participating in surf PE attained HRs and durations that are consistent with recommendations with cardiovascular fitness and health
[39]	Quantitative	Well-being, Physical and Mental Health Education	190 (110 males) Age: 10–13	Surfing as part of the PE curriculum can be considered a safe activity, an important mediator for making new friends and being part of a group with important short-term effects on self-confidence
[23]	Qualitative	Well-being, Physical and Mental Health	22 Age: 9–13	Sailing participation improves personal feelings of confidence and competence; key personal and interpersonal skills, specific life skills, sailing-specific technical skills; physical fitness; and good general mental health
[40]	Qualitative	Education	Unknown Age: 14–15	The students develop basic movement skills for bodyboarding and are introduced to the knowledge of a healthy related physical activity
[13]	Mixed methods Qualitative/Quantitative	Well-being, Physical and Mental Health	595 Age: 6–16	There is clear evidence on the social benefits for school-age children and adolescents associated with participation in outdoor activities in blue spaces: mental health and well-being, education, active citizenship, social behavior, and environmental awareness
[41]	Qualitative	Education	Unknown Age: 9–12	They have gained insight on how to implement a life skill focus within surf-based programs and connected research to practice by shedding light on how to utilize the implicit/explicit continuum
[42]	Qualitative	Education	16 Age: 14–16	Young men were viewed as being more legitimate participants and regularly took up the lead role of skipper—young women were considered secondary participants
[43]	Quantitative	Management	350 Age: 6–12	The results show psychological commitment as one indicator of satisfaction and word of mouth as the only valid indicator to measure behavioral intentions

Source: Own elaboration.

## Data Availability

Data sharing is not applicable.

## Supplementary Materials

The following supporting information can be downloaded at: https://www.mdpi.com/article/10.3390/bs13110905/s1. Table S1. Analysis of the methodological quality of the examined articles. Table S2. Analysis of the methodological quality of the examined articles.
